# Evaluating chemotherapy-driven placental alterations and their impact on fetal development

**DOI:** 10.3389/ftox.2025.1688641

**Published:** 2026-02-11

**Authors:** Katrien De Clercq, Carolina Velazquez, Eleonora Persoons, Vera Wolters, Marieke Van de Ven, Frédéric Amant

**Affiliations:** 1 Implantation, Placentation and Pregnancy (POPPY) Research Group, Department of Development and Regeneration, Katholieke Universiteit Leuven (KU Leuven), Leuven, Belgium; 2 Department of Oncology, Katholieke Universiteit Leuven (KU Leuven), Leuven, Belgium; 3 Department of Gynecologic Oncology, Netherlands Cancer Institute (NKI), Amsterdam, Netherlands; 4 Mouse Clinic for Cancer and Aging (MCCA), Preclinical Intervention Unit, Netherlands Cancer Institute (NKI), Amsterdam, Netherlands; 5 Department of Obstetrics and Gynecology, Universitair Ziekenhuis Leuven (UZ Leuven), Leuven, Belgium

**Keywords:** placenta, chemotherapy, pregnancy, contrast-enhanced microtomography, fetal-toxicity

## Abstract

**Background:**

Chemotherapy during pregnancy presents a clinical challenge, balancing maternal treatment efficacy with fetal safety. While chemotherapy after the first trimester is generally considered safe, its impact on placental development remains underexplored. This study investigates the effects of commonly used chemotherapeutic agents (CAs), including anthracyclines, taxanes, and platinum-based compounds, on maternal, placental, and fetal outcomes using a mouse model.

**Methods:**

To model the chemotherapy exposure during pregnancy, pregnant mice received a single CA dose at embryonic day 13.5 (E13.5), equivalent to the beginning of the second trimester in human gestation. Placental and fetal outcomes were assessed at E15.5 and E18.5 using contrast-enhanced microtomography (micro-CT) and histopathological analyses to investigate the alterations associated to the exposure to differen CAs.

**Results:**

Platinum-based agents, particularly carboplatin, significantly reduced fetal and placental weights and altered placental morphology, with persistent effects observed at E18.5. Contrast-enhanced microtomography (micro-CT) and histopathological analyses revealed reduced placental volumes, in both the labyrinth and junctional zones, and increased signs of trophoblast degeneration. Despite these changes, embryonic viability and litter size remained unaffected, suggesting that fetal growth restriction may be driven by placental insufficiency rather than direct fetal toxicity.

**Conclusion:**

These findings underscore the importance of placental assessment in evaluating chemotherapy safety during pregnancy and highlight the potential long-term implications of platinum-based treatments on fetal development.

## Introduction

The estimated incidence of cancer diagnosed during pregnancy is one per 1,000 pregnancies (de Haan et al., 2018), with breast cancer, cervical cancer and lymphomas the most common cancer types. Different combinations of chemotherapeutic agents (CA) such as anthracyclines, alkylating agents, taxanes, alkaloids or antitumor antibiotics are typically part of the management of these patients. Yet, treating cancer during pregnancy poses a clinical conundrum of optimizing maternal health outcomes while safeguarding fetal wellbeing. Based on reassuring short-term neonatal and pediatric outcomes, chemotherapy administered from the 12th week of pregnancy, after embryonic organogenesis is completed, is considered safe (Silverstein et al., 2020; Van Assche et al., 2023; Vandenbroucke et al., 2020). However, since placental development continues during the second and third trimester in order to comply to the needs of the growing fetus, CA may negatively impact placental development. In this regard, literature states that placentas exposed to CA have a smaller size, poor vascularization and increased DNA damage (Wolters et al., 2021). Even for CA with limited transplacental transport, an increased risk of stillbirth, preterm delivery, and fetal growth restriction (FGR) have been reported (de Haan et al., 2018; Van Calsteren et al., 2010). These observations thus suggest that, to some extent, impaired placental functioning might contribute to CA-associated FGR. In line herewith, FGR in the general population is also primarily caused by placental dysfunction, which renders placental nutrient transport to the fetus insufficient. Importantly, the impact of a malfunctioning placenta extends beyond the fetal period, potentially representing the foundation for life-long health of both the offspring and mother (Bena et al., 2021). Thus, aberrant placentation not only has immediate consequences for pregnancy outcomes, but also predisposes the offspring to metabolic, cardiovascular and neuropsychiatric disorders. It is therefore important to understand how CA affect placental development.

Preclinical models allow for simplification of the clinical setting by interrogating single agents. Moreover, the effects on placental and other fetal or maternal organs can be explored at different time points after the administration. Previous research using a mouse model found substantial variation in transplacental transfer of CA during pregnancy, with high transfer for carboplatin, limited transfer for anthracyclines and no transfer for paclitaxel (Van Calsteren et al., 2011). However, the effect of CA on placental morphology was not determined. Importantly, both mouse and human placentas are hemochorial, meaning that placental trophoblast cells are in direct contact with maternal blood, and, therefore, exposed to CA. In mice, the placenta comprises three distinguishable structures: *i.e.*, the maternal decidua, the junctional zone and the labyrinth zone, where the juxtaposition of maternal and fetal circulations allows for nutrient exchange. Placental function directly arbitrates fetal growth and defects in the formation of any of the placental layers can result in placental insufficiency that, ultimately, can affect fetal development.

In this study, we sought to determine the effect of CA administered during pregnancy on maternal, placental and fetal outcomes, at different time-points, without the presence of confounding factors that are present in the clinic, including cancer type or CA regimen. To do so, the most commonly used CA in cancer during pregnancy were administered as a single dose at E13.5, when organogenesis is completed, and effects at maternal, placental and fetal level were assessed 2 days (E 15.5) and 5 days (E 18.5) after the administration, as a proxy of short-term and long-term, respectively.

## Materials and methods

### Animals

All mice (C57B6/6JRj) were kept in a controlled environment (21 °C ± 1.0 °C, 45%–60% relative humidity, 12:12 h light-dark cycle). Animals were housed in Individually ventilated cages (IVC) cages, maximum five animals per cage with food and water *ad libitum.* Mice were bred at 8 weeks of age and checked daily for the presence of a copulation plug within the vaginal canal. The day of plug detection was dated as embryonic day 0.5 (E0.5). At E13.5, corresponding to the transition of first to second trimester during human pregnancy, mice were injected with a single CA via intravenous tail vein injection when considered pregnant, *i.e*., a 20% increased body weight. At E15.5 or E18.5, mice were sacrificed using CO_2_ and placental and fetal tissues were collected and weighted. Placental efficiency was defined as the ratio of fetal weight to placental weight. Therefore, assessments were done at two different timepoints: 2 days after the CA injection (i.e.,: E15.5, short-term effects) and 5 days after the CA injection (i.e.,: E18.5, long-term effects).

Human endpoints were applied according to the Code of Practice Animal Experiments in cancer research, which included weight loss (*i.e.*, more than 15% of bodyweight within 2 days after intervention, or more than 20% since the start of the experiment), circulation or breathing problems, or aberrant behavior. Maternal organs (kidney, small intestines and ovaries) were collected for toxicity evaluation. Animals with litter size less than 4 were excluded. All experiments were performed under the ethical approval AVD3010020198564 WP 21.1.8924 and 21.1.10032.

The dosage of CA was based on previous studies (Van Calsteren et al., 2011): Doxorubicin 9 mg/kg, Epirubicin 11 mg/kg, Paclitaxel 10 mg/kg, Carboplatin 50 mg/kg, Cisplatin 6 mg/kg. NaCl served as vehicle.

### Contrast enhanced CT

Placentas were fixed in Ethanol-Acidic acid-formalin (EAF), stored in PBS and transferred to Zirconium-substituted Keggin polyoxometalate (Ly and Parac-Vogt, 2017) ((Et_2_NH_2_)_10_[Zr(PW_11_O_39_)_2_]•7H_2_O) (Zr-POM, 35 mg/mL PBS) for 7 days. Placentas were scanned in PBS using a Phoenix Nanotom M (GE Measurement and Control Solutions, Germany) at 4.5 µm isotropic voxel size. The source, equipped with a tungsten target, was operated at 60 kV and 240 μA. An aluminium filter of 0.1 mm was applied to reduce beam hardening. For each sample, 2,400 frames were acquired over 360° using the fast scan mode with an exposure time of 500 m (frame averaging = 1 and image skip = 0) (25), resulting in a scanning time of only 20 min per sample. Afterwards, scan optimization (projection filter, inline volume filter, and beam hardening correction) was applied during 3D reconstruction (Datos|x, GE Measurement and Control solutions).

### CE-CT image processing and analysis

Whole placentas - Images were analyzed using CTAn (Bruker MicroCT). On the dataset, a region of interest (ROI) was drawn manually on every 100th image incorporating the whole placenta. A global threshold and ROI shrink-wrap analysis was used to exclude background noise. Additionally, noise was removed by a closing operation (round kernel, radius 2, 3D space) and a double despeckling step (removal of black and white speckles respectively, 3D space, less than 100 voxels). Using 3D analysis, the placental volume was calculated. CTVox (Bruker MicroCT) was used for 3D visualization of the placentas.

Individual layers - To assess the volume of the different placental layers, a new ROI was drawn manually incorporating only the labyrinth, the junctional zone or the decidua. Using a global threshold, the images were binarized, and noise was removed as described previously. Using 3D analysis, the volume was calculated.

### Histopathology

Mouse organs and embryos were carefully harvested and fixed in either EAF fixative (ethanol/aceticacid/formaldehyde/saline at 40:5:10:45 v/v) or formalin. After fixation, the different organs and embryos were embedded in paraffin.

Two- and four-µm thick sections were prepared and followed by hematoxylin and eosin (H&E) staining, according to standard procedures to assess general morphology and pathological features in the different organs.

For the toxicologic pathology study after therapeutics, the embryos and placentas were prepared in sagittal-sectioning that gave rise to a presentation of embryonic organs/tissues to the greatest extent in one section. For a fully developed placenta (after E12.5), which is composed of three well-defined layers of chorionic plate, labyrinth, and spongiotrophoblasts/giant cells, the sagittal sections showed all three layers in a clear order. Toxicologic pathology study was conducted at macroscopic as well as microscopic levels. Macroscopically, any changes in weight, organ size, color, and appearances of necrosis and/or hemorrhages, etc. Were carefully documented for both embryos and placentas. Microscopically, any alterations in the thickness of an individual layer of placenta and any detections of pathologic features such as apoptosis, degeneration, pyknosis, necrosis, hemorrhages, and/or reduced cellularity, etc. in organs/tissues of embryos and placenta were annotated in detail, from which reports were made.

The sections were blindly reviewed by an animal pathologist under a Zeiss Axioskop2 Plus microscope (Carl Zeiss Microscopy, Jena, Germany) and representative images were captured with a Zeiss AxioCam HRc digital camera and processed with AxioVision 4 software (both from Carl ZeissVision, München, Germany).

### Data display and statistics

Graph display and statistics were done in Graphpad Prism (Graphpad Software, United States). Normality was assessed with D’agostino & Pearson omnibus normality test. Differences were considered statistically significant when p < 0.05. Specific statistical test are mentioned in the figure legends. Statistics were performed on all data for the pilot study, and on litter means for the validation study. Data display and 3D rendering of the CE-CT images was done by Dataviewer and CTan (Bruker MicroCT).

## Results

### Chemotherapy causes compound-specific maternal, fetal and placental toxicity

To evaluate the effect of CAs during pregnancy, mice were administered with a single CA at E13.5, after organogenesis has been completed. The general toxicity of a single dose of CA administration was assessed in three different entities; mother, placenta and fetus; at two different time points; 2 days after the injection (E15.5, short term), and 5 days after the injection (long term, E18.5). First, the effects of the CA administration were assessed on the pregnant mice. Maternal organs, and more specifically the ovaries and small intestines, were affected in all CA groups at E15.5 (Supplementary Table S1). Toxicity in small intestines, measured as level of apoptosis in the crypts, was overall restored at E18.5 (Supplementary Table S2). On the contrary, the level of apoptosis in preantral follicles of the ovaries was retained at E18.5.

**FIGURE 1:**
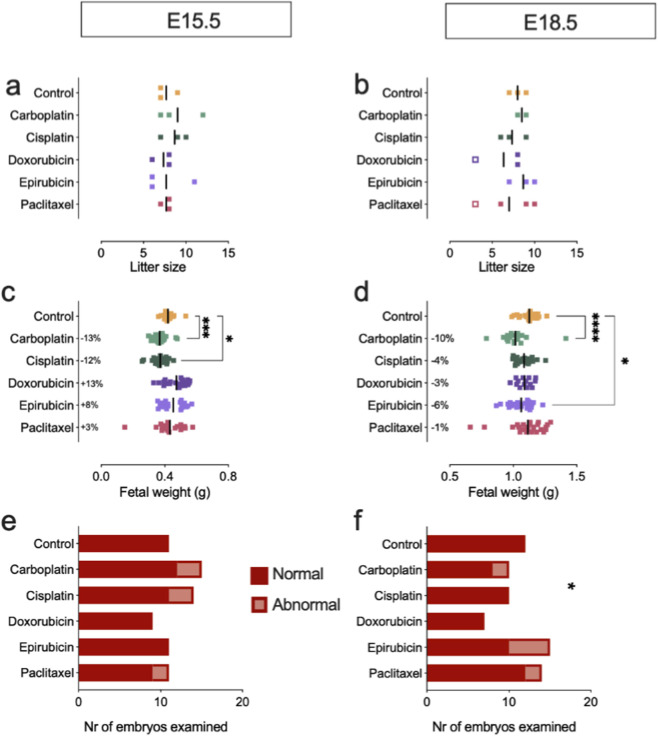
Effect of CA on fetal outcome. **(a)** Litter size at E15.5. **(b)** Litter size at E18.5. open squares show litters that were excluded for analysis. **(c)** Fetal weight at E15.5. **(d)** Fetal weight at E18.5. n = 17–27 fetuses from N = 3-4 litters. **(e)** Toxicological evaluation of fetus at E15.5. **(f)** Toxicological evaluation of embryo at E18.5. n = 6–15 from N = 3-4 litters **(c,d)**: Kruskal-Wallis test with Dunn’s multiple comparison test, compared to control group. **(e,f)**: Fisher’s exact test. Data are shown as individual points and mean.

Second, the CA administration effect on the embryos was evaluated (Table 1). Litter size and viability was not affected at any of the time points by CA administration (Figures 1a,b). In contrast, effects on fetal weight were compound dependent. Specifically, treatment with carboplatin significantly reduced fetal weight both at E15.5 and E18.5, whereas fetal weight was significantly lower at E15.5, but not at E18.5 after treatment with cisplatin (Figures 1c,d). Epirubicin treatment caused fetal weight reduction at E18.5, associated with a numerical increase in the proportion of embryos that presented signs of toxicity, *i.e.*, degeneration of the liver. Fetal weight was unaffected in doxorubicin and paclitaxel treated animals. Moreover, no significant embryonic toxicity was reported after treatment with carboplatin, cisplatin, doxorubicin and paclitaxel at either E15.5 or E18.5 (Figures 1e,f; Supplementary Tables S3–S4).

**TABLE 1:** Overview of effect on fetal and placental variables on short term (ST) and long term (LT).

Exposure	Control		Carboplatin		Cisplatin		Doxorubicin		Epirubicin		Paclitaxel	
	ST	LT	ST	LT	ST	LT	ST	LT	ST	LT	ST	LT
Litter size (median)	**7**	**8**	**8**	**8.5**	**9**	**7**	**8**	**8**	**6**	**9**	**8**	**7.5**
Fetal weight (mg)	**420.0**	**1,126**	**367.0**	**1,017**	**371.1**	**1,085**	**472.8**	**1,088**	**452.1**	**1,062**	**431.4**	**1,115**
Placental weight (mg)	**177.5**	**150.2**	**156.8**	**123.3**	**149.3**	**150.5**	**160.4**	**141.0**	**160.5**	**138.1**	**152.0**	**147.1**
Placental volume (mm3)	**91.25**	**78.88**	**78.63**	**73.07**	**79.95**	**75.62**	**71.93**	**71.23**	**80.39**	**69.47**	**80.14**	**74.68**
Labyrinth volume (mm3)	**36.91**	**36.28**	**32.17**	**34.57**	**32.22**	**32.30**	**28.75**	**29.25**	**31.85**	**32.07**	**33.07**	**34.70**
Junctional zone volume (mm3)	**30.74**	**19.90**	**25.14**	**16.47**	**27.06**	**19.63**	**22.96**	**15.93**	**26.06**	**16,12**	**26.63**	**17.32**

Fetal variables litter size (measured as a number of number of pups born to a mother in one birth) and fetal weight at birth (measured in mg) together with placental variables as placental weight (measured in mg) with the different placental-related volumes measured in mm3.

**FIGURE 2:**
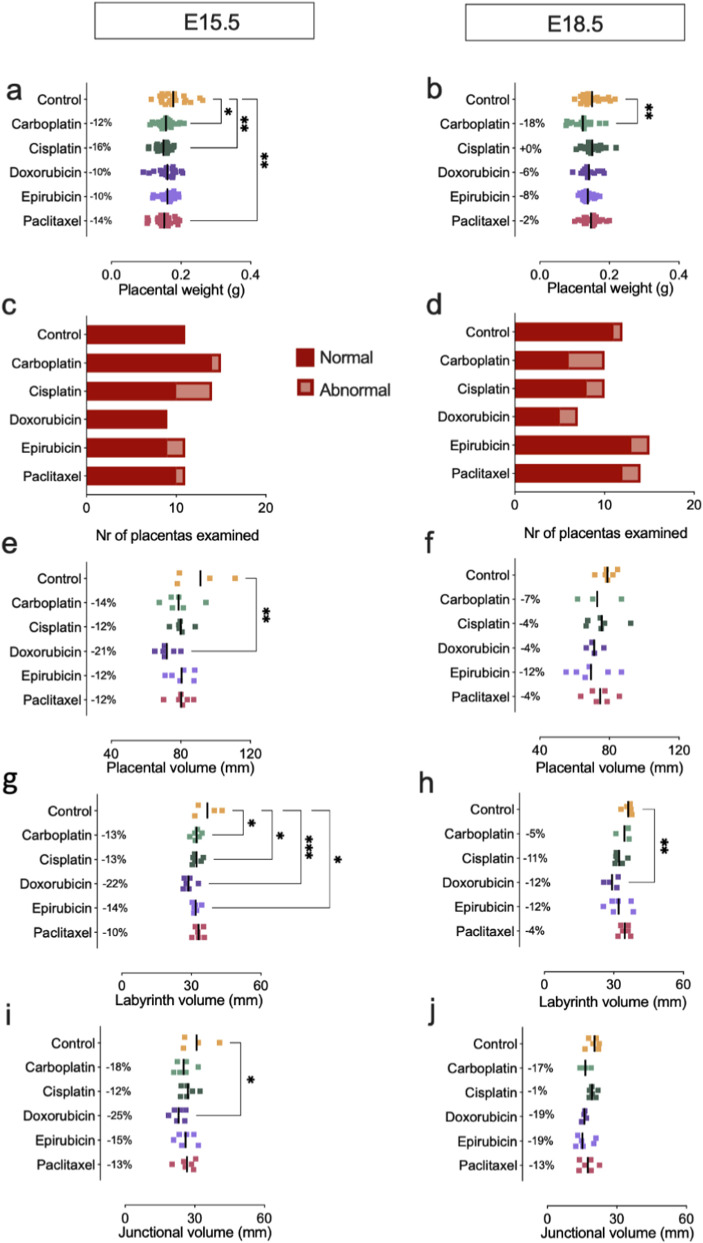
Effect of CA on placenta. **(a)** Placental weight at E15.5. **(b)** Placental weight at E18.5. n = 17–27 embryos from N = 3-4 litters **(c)** Toxicological evaluation of placenta at E15.5. **(d)** Toxicological evaluation of embryo at E18.5. n = 6–15 from N = 3-4 litters **(e)** Placental volume at E15.5. **(f)** Placental volume at E18.5. **(g)** Labyrinth volume at E15.5. **(h)** Labyrinth volume at E18.5. **(i)** Junctional zone volume at E15.5. **(j)** Junctional zone volume at E18.5. n = 4-6 from N = 3-4 litters. **(a,b)**: One-way ANOVA with Dunnett’s multiple comparison test, compared to control group. **(c–d)**: Fisher’s exact test. **(e-j)**: One-way ANOVA with Dunnett’s multiple comparisons test. Data are shown as individual fetuses/placentas and mean.

Third, placentas were inspected in order to detect signs of toxicity associated with any of the administered CA (Table 1). Placental weight at E15.5 was reduced for all CA groups by at least 10% (10%–16%), and significantly when treated with platins and taxanes (Figure 2a). While this reduction was not observed anymore at E18.5 for cisplatin or paclitaxel, fetal weight after carboplatin treatment was not restored on long term (Figure 2b). An increase in the proportion of placentas with signs of toxicity was observed at both E15.5 or E18.5, albeit not significant (Figures 2c,d; Supplementary Tables S5–S6). Placental efficiency, defined as the grams of fetus produced per gram placenta (FW:PW ratio), was calculated. In general, the values were comparable to the control group but, interestingly, a transient increase in placental efficiency was observed at E15.5 for anthracyclines and taxanes treatment and at E18.5 for carboplatin (Supplementary Figures S1a–b). At E15.5, both anthracycline and taxane treatments were associated with limited fetal weight loss, alongside higher placental efficiency. At E18.5, after carboplatin administration, a relative increase in placental efficiency was observed but this was insufficient to warrant the expected fetal weight.

Furthermore, to assess the gross morphology of the placentas, placental volumes were quantified with CE-CT. Placental volumes at E15.5, determined by contrast-enhanced microCT, were reduced after CA treatment by at least 12% (12%–21%), and significantly after treatment with doxorubicin (Figure 2e). This decrease was still notable at E18.5, albeit not significantly (Figure 2f). Volume reduction at E15.5 was mostly attributed to reductions in the volume of the labyrinth, by at least 10% (10%–22%) (Figure 2h). On top of affecting the volume of the labyrinth, doxorubicin treatment also significantly reduced junctional zone volumes. The labyrinth and junctional zone volumes remained smaller at E18.5, though not significantly or disproportionally (Supplementary Figures S1c–d).

Collectively, in this pilot study, pregnant mice treated with a single dose of CA presented with signs of toxicity in short term, that were resolved in the long term. However, treatment with platinum compounds, especially carboplatin, resulted in a significant and retained toxic effect in the different assessed entities. Importantly, embryonic viability and general development were not affected suggesting that the dosages used were compatible with pregnancy.

### Placental growth is affected when treated with platinum-based CA

**FIGURE 3:**
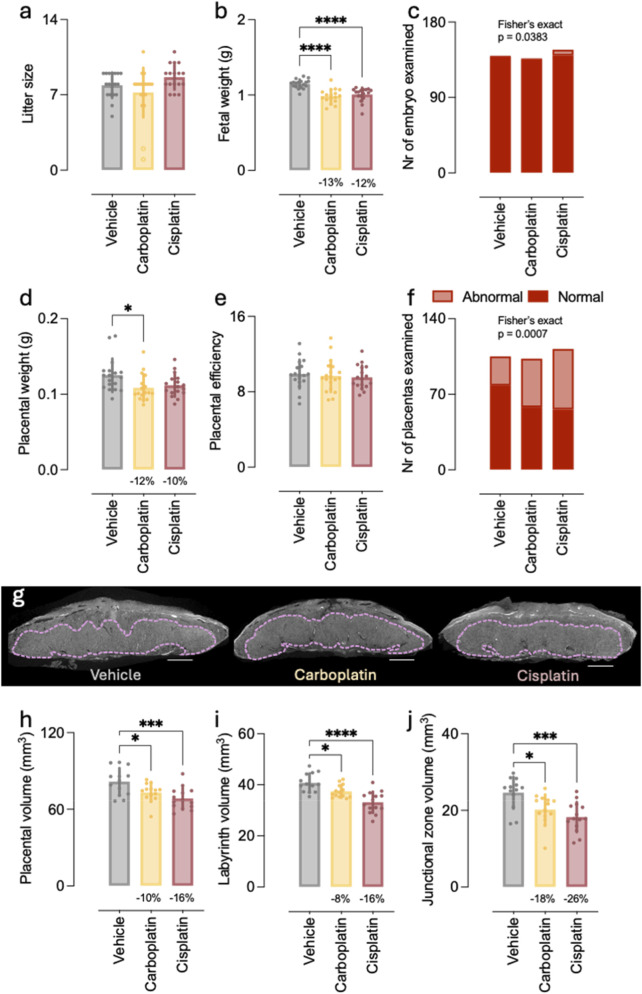
Effect of platins on fetal and placental outcomes at E18.5. **(a)** Litter size. **(b)** Fetal weight. **(c)** Toxicological evaluation of embryo, n = 137–140 fetuses. **(d)** Placental weight. **(e)** Placental efficiency. **(f)** Toxicological evaluation of embryo, n = 103–112 placentas. **(g)** Representative image of CE-CT with labyrinth annotated. Scale bar = 500 um. **(h)** Placental volume. **(i)** Labyrinth volume. **(j)** Junctional zone volume. b, d, i Kruskal-Wallis test with Dunn’s multiple comparisons test. e, g, h One-way ANOVA with Dunnett’s multiple comparisons test. c, **(f)** Fisher’s exact test. **(A,B,D,E)** data are shown litter mean ± SD from n = 17–18 litters. **(G–I)** data are shown individual samples and mean ± SD from n = 13–14 litters.

Large cohort studies in the pregnant cancer population reported FGR in up to 21% of patients receiving chemotherapy. Importantly, platinum agents showed the highest odds ratio (OR for FGR 3.12, 95% CI 1.45–6.70) (de Haan et al., 2018). These findings, together with our results using this mouse model, prompted us to perform a more in-depth exploration of the placental and fetal long-term toxicity of platin-based CA. According to the same experimental setup, mice were administered a single dose of either cisplatin or carboplatin at E13.5 of pregnancy and fetal and placental outcomes were assessed at E18.5 (Table 2). Treatment with cisplatin, and to a lesser extent carboplatin, caused a significant increase in the proportion of mothers presenting with toxicity, restricted to the ovaries and/or kidney without affecting the small intestines (Supplementary Figure S2a; Supplementary Table S7). Moreover, maternal body weight at E18.5 was significantly lower, indicating that gestational weight gain was affected by CA treatment (Supplementary Figure S2b). Given that litter size was not affected (Figure 3a), gestational weight gain adjusted for litter size was lower in CA treated groups compared to vehicle (Supplementary Figures S2c–d). As such, mothers treated with carboplatin and cisplatin gained on average 4.1 g and 3.9 g per fetus, respectively, compared to vehicle treated mothers that gained on average 4.9 g per fetus (p < 0.01).

**TABLE 2:** Overview of effect on fetal and placental variables of platin-based chemotherapy during pregnancy.

Exposure	Control	Carboplatin	Cisplatin
Litter size (median)	**8**	**8**	**9**
Fetal weight (mg)	**1,147**	**985.9**	**1,007**
Placental weight (mg)	**125**	**108.5**	**117.7**
Placental volume (mm3)	**81.48**	**72.96**	**68.25**
Labyrinth volume (mm3)	**40.72**	**37.39**	**33.12**
Junctional zone volume (mm3)	**24.58**	**20.17**	**18.24**

Fetal variables litter size (measured as a number of number of pups born to a mother in one birth) and fetal weight at birth (measured in mg) together with placental variables as placental weight (measured in mg) with the different placental-related volumes measured in mm3.

In line herewith, fetal weight was significantly reduced at E18.5 in carboplatin and cisplatin treated mice, compared to vehicle (0.98 ± 0.09 g (−13%), 1.00 ± 0.09 g (−12%) vs. 1.15 ± 0.06 g, respectively) (Figure 3b). Cisplatin treatment caused toxicity in merely 5% of the evaluated embryos, which was mostly restricted to one organ such as the ovary or testis (Figure 3c; Supplementary Table S8), while no embryonic toxicity signs were observed for carboplatin.

**FIGURE 4:**
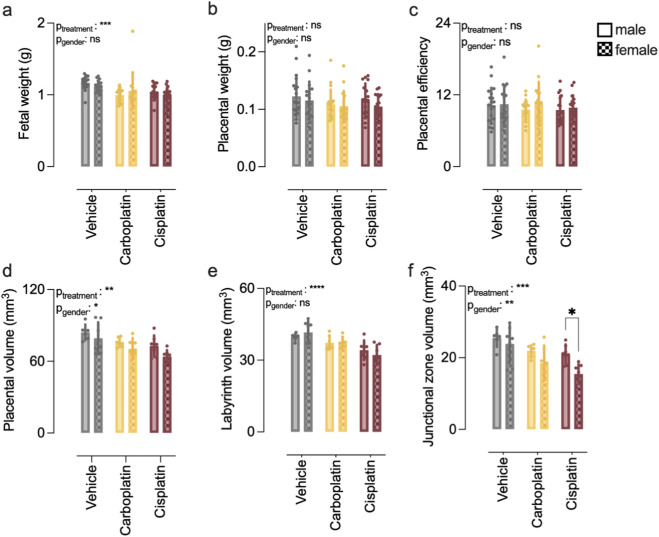
Gender-specific effects of platins on fetal and placental outcomes. **(a)** Fetal weight. **(b)** Placental weight. **(c)** Placental efficiency. **(d)** Placental volume. **(e)** Labyrinth volume. **(f)** Junctional zone volume. Two-way ANOVA with Sidak’s multiple comparisons group, data are shown from individual fetuses and mean ± SD from n = 12–13 litters, with at least 6 female and males.

Although placental weight was numerically reduced by at least 10% in both CA-treated groups, the reduction was only statistically significant for those treated with carboplatin (Figure 3d). An unaltered placental efficiency for both cisplatin and carboplatin suggested that fetal growth was still in concordance with placental capacity (Figure 3e). Nonetheless, significantly larger proportions of placentas showed signs of toxicity, affecting 44% and 50% of examined placentas in the carboplatin and cisplatin treated group, respectively, compared to 25% in vehicle treated group (Figure 3f). Similar to the vehicle treatment, the most commonly reported observation was pyknosis in the labyrinth, suggestive for necrosis or apoptosis (Supplementary Table S9). The assessment of the gross morphology of the placentas by quantifying the placental volumes using CE-CT (Figure 3g) showed that the overall placental volume was significantly reduced in both carboplatin and cisplatin groups by at least 10% (Figure 3h). Since both labyrinth and junctional zone volumes were equally affected (Figures 3i,j), the labyrinth–to–junctional zone ratio was unaltered in the CA-treated placentas compared to vehicle (Figure 3e). These findings suggest that CA with platinum-based compounds affected placental growth in general. Moreover, fetal sex had an overall effect on placental volume and junctional zone volume (Figure 4), being the cisplatin-induced reduction on junctional zone volume significantly worse in female compared to males.

## Discussion

Increasing numbers of women are being diagnosed with cancer during pregnancy, mostly explained by the trend to delayed conception. Consequently, the use of CA, as part of their clinical management, during pregnancy increases as well (de Haan et al., 2018; Maggen et al., 2020; Amant et al., 2015). When administered after the 12th week of pregnancy, chemotherapy is overall well tolerated with limited side effect for the fetus of pregnancy. However, significantly higher incidence of up to 21% of FGR was observed (Maggen et al., 2022). While FGR is often related to placental dysfunction, it remains poorly understood to which extent CA affect the placenta. Hence, given the increased use of CA during pregnancy (de Haan et al., 2018) and its potential to adversely affect placenta (Yu et al., 2024), it is essential to investigate whether CA can contribute to placental dysfunction and consequently determine fetal outcomes.

Using a mouse model (Schuurman et al., 2023), the effects of the most commonly used CA during pregnancy (anthracyclines, cyclophosphamide, taxanes, platinum-based agents) were evaluated on maternal, fetal and placental outcomes both in the short and long term after administration. The model provided the opportunity of 1) investigate individual agents, 2) eliminate confounding factors such as cancer type and maternal conditions, and importantly 3), assess three different entities, i.e., mother, placenta and fetus, at two different time-points. Overall, the low incidence of congenital abnormalities and the unaffected litter size across all CAs support the use of this model for studying the effects of chemotherapy during pregnancy. It should be noted, however, that gestational weight gain was reduced following CA administration, particularly in animals treated with platinum-based agents. Similarly, reduced gestational weight gain has been reported in pregnant women who received chemotherapy, and FGR was diagnosed in those experiencing the least weight gain (Maggen et al., 2022). This finding indicates that gestational weight gain might be a confounding factor when assessing fetal weight and should be taken into account.

While no significant embryonic toxicity was observed for none of the CA, a compound-dependent effect on placenta and fetal weight was observed. Importantly, evaluating the outcomes at two different time points after CA administration allowed us to determine whether the effects were more immediate and whether they were restored preterm. While doxorubicin treatment did not cause significant fetal or placental weight reduction either in short or long term, epirubicin, another anthracycline, did affect fetal weight in long term (Soininen et al., 2015). Paclitaxel reduced placental weight in short term without affecting fetal weight. On the contrary, carboplatin significantly decreased fetal and placental weight already 2 days after the administration which was not restored preterm. In contrast, cisplatin reduced fetal and placental weight in short term, but not in long term, suggesting recovery over time after the single injection *in utero*. These results indicate that the administration of platinum compounds in particular was associated with a lower fetal weight that was retained long term. This observation is consistent with the low weight percentiles of children born to women receiving CA during pregnancy (Maggen et al., 2022), especially for those receiving platinum-based chemotherapy during pregnancy (de Haan et al., 2018).

Given the high transplacental passage of platinum-compounds, fetal growth deficits are assumed to be caused by the impact on fetal development. However, whether placental defects contribute to platinum-associated FGR is unknown. Therefore, the effect of platinum compounds on placental dysfunction and its potential causality for FGR was further validated. Indeed, *in utero* platinum-based CA exposure reduced both gestational weight and prenatal fetal weight compared to the vehicle group. Fetal and placental weight were equally affected, suggesting that the fetus still grows according to the provision of the placenta, and not disproportionally less than what is provided. In line herewith, no toxicity was observed in the embryo.

These findings, together with the fact that no intestinal toxicity was observed in the mothers, suggest that fetal weight decrease might be caused placental insufficiency.

Morphological assessment of the placentas confirmed that the volumes were decreased, affecting both labyrinth and junctional zone and therefore the relative density of the placental layers was preserved. Furthermore, most of the histologically assessed toxicity was observed in the labyrinth, which is responsible for nutrient exchange, suggesting that a single dose of platinum-based CA may affect placental growth and transport capacity, probably resulting in a proportional fetal weight decrease.

Although the toxicity assessment on the maternal and embryo tissue was mainly exploratory and some of the toxicity signs were cleared up in the long-term evaluation, the observations are similar to intestinal and germ-cell toxicity described in patients treated with chemotherapy. The intestinal epithelium is rich in rapidly dividing cells and hence is a prime target for CA, explaining the gastrointestinal expected treatment side effects as nausea, constipation, vomiting, diarrhea, abdominal pain, weight loss and ulcerations (Akbarali et al., 2022). This, in turn, may limit food intake and indirectly affect gestational weight gain. Moreover, it is also known that the ovarian follicle reserve is extremely sensitive to the effects of CA (Spears et al., 2019). The toxicity observed in the ovarian follicles and the persistent elevated apoptosis emphasizes the repercussion of chemotherapy on premature ovarian insufficiency and infertility observed in cancer survivors (Mauri et al., 2020). Remarkably, the fertility-related toxicity observed in the embryos in both ovarian follicles and testis underscores the importance of monitoring the normal sexual development and fertility in the children exposed to chemotherapy *in utero*.

Importantly, the mouse model allowed us to explore the CA-related effect on placenta and embryos preterm, which is not feasible in the clinical setting. These important results reveal that CA, specifically platinum compounds, may impact the growth and transport capacity of placenta, thereby compromising fetal outcomes. However, the applied approach cannot capture the complexity that is associated with treatment in the clinics. The fact that only a single dose was administered impedes the possibility to assess cumulative toxicity. This could potentially underestimate the toxicity signs observed at long term. Moreover, the strategy of studying single agents does not allow for studying the synergistic effects of combinatory CA exposures. This is of importance given that patients usually receive multiple administrations of a cocktail of different chemotherapeutic compounds that potentiate the cytotoxicity.

## Data Availability

The original contributions presented in the study are included in the article/Supplementary Material, further inquiries can be directed to the corresponding authors.
