# Supplementary material for: Evaluating chemotherapy-driven placental alterations and their impact on fetal development
Source: Front Toxicol. 2026 Feb 11;7:1688641. doi: 10.3389/ftox.2025.1688641 (PMC12932932; doi:10.3389/ftox.2025.1688641)
Supplement: Supplementary file 1 [file DataSheet1.docx]

**Evaluating chemotherapy-driven placental alterations and their impact on fetal development**

**Katrien De Clercq**^1,*^, Carolina Velazquez^2^, Eleonora Persoons^1^, Vera Wolters^3^, Marieke van de Ven^4^, Frédéric Amant^2,3,5*^

^1^Implantation, Placentation and Pregnancy (POPPY) Research Group, Department of Development and Regeneration, KU Leuven, Herestraat 49 box 611, 3000 Leuven, Belgium

^2^Department of Oncology, KU Leuven, Leuven, Belgium

^3^Gynecologic Oncology, Netherlands Cancer Institute, Amsterdam, Anthony Van Leeuwenhoek, The Netherlands

^4^Mouse Clinic for Cancer and Aging (MCCA), Preclinical Intervention Unit, Netherlands Cancer Institute, Amsterdam, The Netherlands

^5^Department of Obstetrics and Gynecology, UZ Leuven, Leuven, Belgium

^*^Corresponding authors:

Prof. dr. Katrien De Clercq

Implantation, Placentation & Pregnancy (POPPY) research group

Department of Development and Regeneration, KU Leuven

Herestraat 49, bus 611

3000 Leuven, Belgium

Email: [Katrien.declercq@kuleuven.be](mailto:Katrien.declercq@kuleuven.be)

Prof. Dr. Frédéric Amant

Experimental Gyneacology

Department of Oncology, KU Leuven

Herestraat 49,

3000 Leuven, Belgium

Email: [Frederic.amant@uzleuven.be](mailto:Frederic.amant@uzleuven.be)

**SUPPLEMENTARY FIGURES AND TABLES**


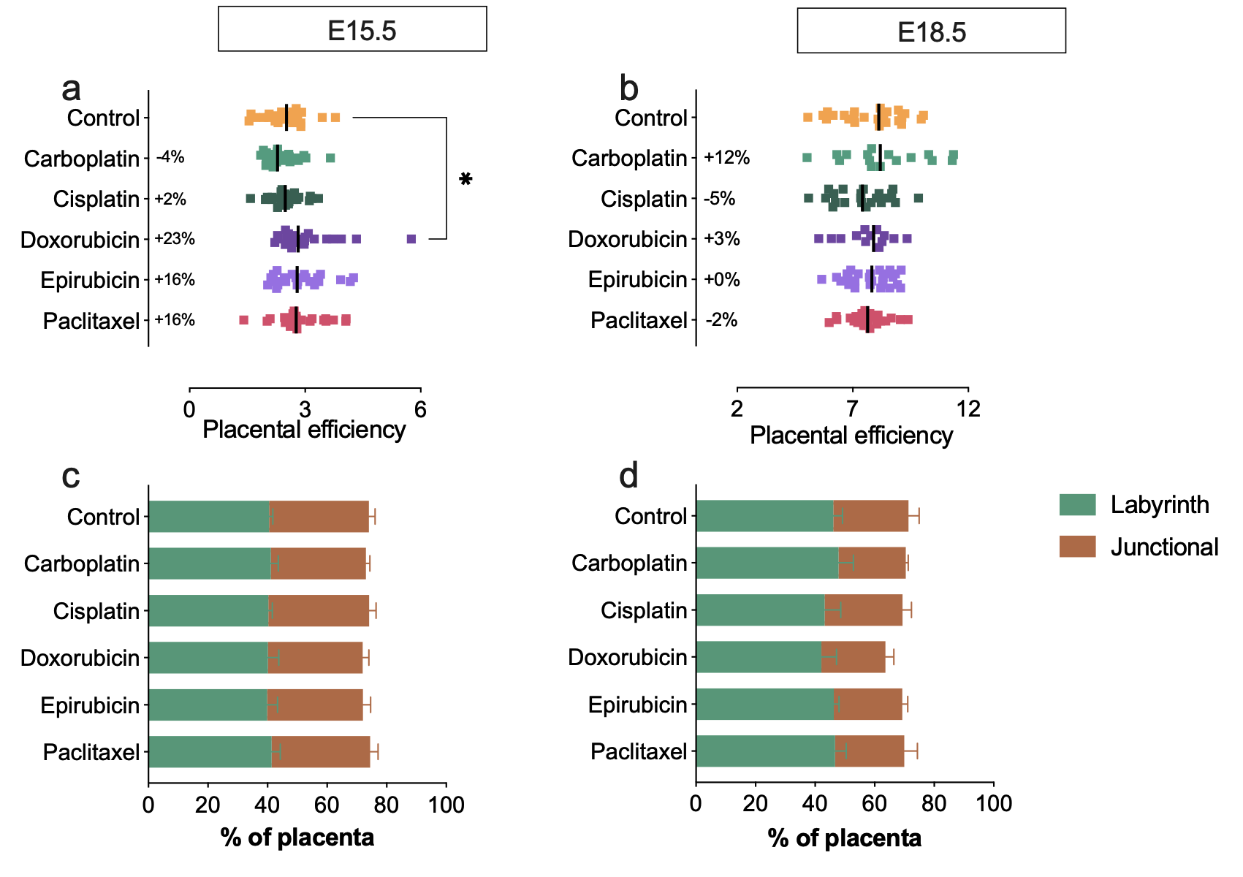


**Figure S1: effect of CA on placental efficiency and density.** (a) placental efficiency on E15.5, Kruskal-Wallis test with Dunn’s multiple comparisons test. (b) placental efficiency on E18.5. n = 17-27 fetuses from N = 3-4 litters (c) Density of labyrinth and junctional zone on E15.5. (d) Density of labyrinth and junctional zone on E18.5. n = 4-6 from N = 3-4 litters.

**Figure S2: Effect of platins on maternal and fetal outcomes.** (a) toxicological evaluation of mothers at E18.5 pregnancy, Fisher’s exact test, n = 15 – 19 mums. (b) maternal weight during pregnancy; E0.5 = day of plug, E13.5 = day of CA administration, E18.5 = day of sacrifice, Two-way ANOVA with Tukey’s multiple comparisons test, n = 20-21 at E0.5, n=9-11 at E13.5, n = 16-17 on E18.5. (c) Correlation between gestational weight gain and litter size. (d) adjusted gestational weight gain, Kruskal-Wallis test with Dunn’s multiple comparisons test, n = 17. (e) Labyrinth – to – junctional zone ratio. (b,d,e) data are shown as individual moms and mean ± SD

**Figure S3: Gender-specific effects of platins on fetal and placental outcomes.** (a) Fetal weight. (b) Placental weight. (c) Placental efficiency. (d) Placental volume. (e) Labyrinth volume. (f) Junctional zone volume. Two-way ANOVA with Sidak’s multiple comparisons group, data are shown from individual fetuses and mean ± SD from n = 12-13 litters, with at least 6 female and males .

|  | Maternal pathology | Ovaries | | | | Small intestines | Kidney |
| --- | --- | --- | --- | --- | --- | --- | --- |
| Group  Pilot E15.5 |  | Absence of follicles | Apoptosis in preantral follicles | Limited apoptosis in preantral follicles | Necrosis (pre)antral follicles | Increased apoptosis in crypts | Local degeneration of nephron |
| Vehicle | 0/3 |  |  |  |  |  |  |
| Carboplatin | 3/3 | 1/3 | 2/3 |  |  | 3/3 | 1/3 |
| Cisplatin | 2/3 |  |  | 2/3 |  | 3/3 |  |
| Doxorubicin | 4/4 |  | 4/4 |  | 1/4 | 3/4 |  |
| Epirubicin | 3/3 |  | 3/3 |  |  | 3/3 |  |
| Paclitaxel | 2/3 | 1/3 |  | 1/3 |  |  |  |

**Table S1: Maternal toxicity 48hrs (E15.5) after exposure to a single CA dose.**

|  | Maternal pathology | Ovaries | | | | Small intestines | | Kidney | |
| --- | --- | --- | --- | --- | --- | --- | --- | --- | --- |
| Group  Pilot E18.5 |  | Absence of follicles | Apoptosis in preantral follicles | Limited apoptosis in preantral follicles | Necrosis (pre)antral follicles | Depletion of the lymphocytic population in Peyer’s patches | Colitis with necrosis of the crypts in proximal colon | Degeneration of the renal epithelial cells | Depletion of the lymphocytic population in renal lymph nodes |
| Vehicle | 0/3 |  |  |  |  |  |  |  |  |
| Carboplatin | 1/2 |  | 1/1 |  |  |  | 1/1 |  |  |
| Cisplatin | 0/3 |  |  |  |  |  |  |  |  |
| Doxorubicin | 3/3 |  | 3/3 |  |  |  |  |  |  |
| Epirubicin | 3/3 |  | 3/3 |  |  |  |  |  |  |
| Paclitaxel | 0/4 |  |  |  |  |  |  |  |  |

**Table S2: Maternal toxicity 96hrs (E18.5) after exposure to a single CA dose.**

| Group  Pilot E15.5 | Embryonic abnormalities | Description |
| --- | --- | --- |
| Vehicle | 0/11 (0%) |  |
| Carboplatin | 3/15 (20%) | - Few apoptotic cells in the seminiferous tubules of testis (2/3)  - Necrosis and pyknosis in liver and in spinal cord. |
| Cisplatin | 3/14 (21%) | - Few apoptotic cells in the seminiferous tubules of testis (3/3) |
| Doxorubicin | 0/9 (0%) |  |
| Epirubicin | 0/11 (0%) |  |
| Paclitaxel | 2/11 (18%) | - Few apoptotic cells in the seminiferous tubules of testis (2/2) |

**Table S3: Pathological assessment of fetuses 48hrs (E15.5) after exposure to CA.**

| Group  Pilot E18.5 | Embryonic abnormalities | Description |
| --- | --- | --- |
| Vehicle | 0/12 (0%) |  |
| Carboplatin | 2/10 (20%) | - Severe edema in the subcutis of trunk.  - Degenerative changes of the liver parenchyma and intestinal epithelia. |
| Cisplatin | 0/10 (0%) |  |
| Doxorubicin | 0/7 (0%) |  |
| Epirubicin | 5/15 (33%) | - Local hemorrhages and degeneration of liver. (5/5) |
| Paclitaxel | 2/14 (14%) | - Pyknotic cells were present in the seminiferous tubules of testis. (2/2) |

**Table S4: Pathological assessment of fetuses 96hrs (E18.5) after exposure to CA.**

| Group  Pilot E15.5 | Placental abnormalities | Description |
| --- | --- | --- |
| Vehicle | 0/11 (0%) |  |
| Carboplatin | 1/15 (7%) | - Apoptosis and pyknosis in the embryonic vessels in Labyrinth. |
| Cisplatin | 4/14 (29%) | - Degeneration of some of the labyrinthine trophoblasts (regional).  - Regional degeneration of labyrinthine trophoblasts as well as spongiotrophoblasts/glycogen cells. |
| Doxorubicin | 0/9 (0%) |  |
| Epirubicin | 2/11 (18%) | - Local necrosis and edema in the area beneath JZ.  - Degeneration of labyrinthine trophoblasts (regional). |
| Paclitaxel | 1/11 (9%) | - Dilation/congestion of the maternal sinusoids in Labyrinth. |

**Table S5: Pathological assessment of placentas 48hrs (E15.5) after exposure to CA.**

| Group  Pilot E18.5 | Placental abnormalities | Description |
| --- | --- | --- |
| Vehicle | 1/12 (8%) | - Degeneration of some of the labyrinthine trophoblasts. |
| Carboplatin | 4/10 (40%) | - Degeneration of some of the labyrinthine trophoblasts (regional).  - Degeneration (pyknosis) of many of the labyrinthine trophoblasts (regional).  - Congestion in labyrinth zone.  - Degeneration of the labyrinth of both embryonic and maternal compartments. |
| Cisplatin | 2/10 (20%) | - Degeneration of some of the labyrinthine trophoblasts. (2/2) |
| Doxorubicin | 2/7 (29%) | - Degeneration of some of the labyrinthine trophoblasts. (2/2) |
| Epirubicin | 2/15 (13%) | - Degeneration (pyknosis) of many of the labyrinthine trophoblasts (regional).  - Congestion in labyrinth zone. |
| Paclitaxel | 2/14 (14%) | - Degeneration of some of the labyrinthine trophoblasts (regional).  - Degeneration (pyknosis) of many of the labyrinthine trophoblasts (regional). |

**Table S6: Pathological assessment of placentas 96hrs (E18.5) after exposure to CA.**

|  | Maternal pathology | Ovaries | | Small intestines | | Kidney | | |
| --- | --- | --- | --- | --- | --- | --- | --- | --- |
| Group  Validation E18.5 |  | Apoptosis in preantral follicles | Limited apoptosis in preantral follicles | Mild lesions of colitis | Local presence of fungus-like organism in the mucosa of jejunum | Focal degeneration of the renal epithelia with proteinaceous casts | Multifocal degeneration of the renal epithelia with proteinaceous casts | Atrophy of the nephrons with local degeneration of the renal epithelia and proteinaceous casts |
| Vehicle | 1/18 | 1/1 |  |  |  |  |  |  |
| Carboplatin | 4/19 |  | 4/4 | 1/4 | 1/4 |  |  |  |
| Cisplatin | 9/17 |  | 5/9 |  |  | 5/9 | 2/9 | 1/9 |

**Table S7: Maternal toxicity 96hrs (E18.5) after exposure to platin treatment.**

| Group  Validation E18.5 | Embryo abnormalities | Description |
| --- | --- | --- |
| Vehicle | 1/149 (0.7%) | - Necrosis (reabsorption) |
| Carboplatin | 1/137 (0.7%) | - Few apoptotic cells in ovary. |
| Cisplatin | 7/147 (5%) | - Necrosis (reabsorption) (1/7)  - Emphysema-like changes in lung (1/7)  - Erythropoiesis with some apoptosis in embryonic ovary (3/7)  - Erythropoiesis in embryonic testes (2/7) |

**Table S8: Pathological assessment of fetuses 96hrs (E18.5) after exposure to platin treatment**

| Group  Validation E18.5 | Placental abnormalities | Description |
| --- | --- | --- |
| Vehicle | 27/105 (25%) | - Pyknotic changes in some of the labyrinthine trophoblasts (23/27)  - Local congestion (2/27)  - Pyknotic changes in some of the spongiotrophoblast (1/27)  - Dilatation of maternal blood sinus (1/27) |
| Carboplatin | 45/103 (44%) | - Pyknotic changes in some of the labyrinthine trophoblasts (43/45)  - Thin labyrinth (2/45)  - Pyknotic changes in some of the spongiotrophoblast (2/45) |
| Cisplatin | 56/112 (50%) | - Pyknotic changes in some of the labyrinthine trophoblasts (51/56)  - Pyknotic changes in some of the spongiotrophoblast (2/56)  - Atrophy of spongiosum (1/56)  - Necrosis (2/56) |

**Table S9: Pathological assessment of placentas 96hrs (E18.5) after exposure to platin treatment**
